# Autoimmune Polyglandular Syndrome Type 3: A Case Report

**DOI:** 10.7759/cureus.76152

**Published:** 2024-12-21

**Authors:** Joana Subtil, Rui Carvalho, Ana Filipa Rebelo, Paulo Subtil

**Affiliations:** 1 Internal Medicine, Centro Hospitalar de Trás-os-Montes e Alto Douro, Vila Real, PRT

**Keywords:** autoimmune gastritis, autoimmune polyglandular syndrome type 3, autoimmune thyroid disorders, type 1 diabetes mellitus (t1d), vitiligo

## Abstract

Autoimmune polyglandular syndromes (APS) are characterized by associations of two or more autoimmune diseases (AID). APS type 3 is characterized by the presence of autoimmune thyroid disease associated with other AID, excluding adrenal gland involvement.

Here we report a case of a 64-year-old male, with history of type 1 diabetes mellitus (T1DM), diagnosed at the age of 32, who was referred to a Diabetes consultation in 2014 due to poor metabolic control. An optimized intensive insulin regimen with basal insulin glargine and aspart insulin at all meals was implemented. During the investigation, he tested positive for anti-glutamic acid decarboxylase (GAD) 65, anti-islet cell (ICA) and anti-insulin antibodies. In 2019, the patient exhibited achromic spots on the chin, bilateral cervical region, and right hip, and was referred to a dermatology consultation, where he was diagnosed with vitiligo. In a follow-up appointment in 2020, a blood test showed macrocytic anemia with vitamin B12 deficiency. An upper gastrointestinal endoscopy revealed chronic pangastritis. Positive anti-gastric parietal cell antibodies and negative anti-intrinsic factor antibodies led to a diagnosis of chronic autoimmune gastritis, and the patient started monthly injectable vitamin B12 supplementation. Thyroid antibodies were tested to screen for other AID, revealing positive anti-thyroid peroxidase antibodies. The patient had slightly elevated thyrotropin (thyroid-stimulating hormone (TSH)) with normal free thyroxine (FT4), indicating subclinical hypothyroidism that did not require supplementation, and remained under surveillance. Adrenocorticotropic hormone (ACTH) and cortisol levels were normal, ruling out adrenal involvement.

This case demonstrates a type 1 diabetic patient additionally diagnosed with vitiligo, chronic autoimmune gastritis, and chronic autoimmune thyroiditis. Excluding adrenal gland involvement, this constitutes a diagnosis of APS3. Type 1 diabetes mellitus alone poses an increased risk of developing other AID, which can be diagnosed in pre-symptomatic stages through the monitoring of specific autoantibodies, highlighting the importance for clinicians to remain vigilant even in the absence of specific symptoms.

## Introduction

Autoimmune polyglandular syndrome (APS) refers to a group of conditions involving multiple autoimmune diseases that affect two or more endocrine glands [1]. In all types of APS, females are more frequently affected than males [1]. APS type 3 (APS3) is a subset of autoimmune polyglandular syndromes characterized by the coexistence of autoimmune thyroid disease with other autoimmune conditions, excluding Addison's disease and hypoparathyroidism [1,2]. Patients with APS3 can exhibit various autoimmune disorders, including vitiligo, alopecia areata, type 1 diabetes mellitus (T1DM), pernicious anemia, and others. Diagnosis usually involves evaluating clinical symptoms and confirming the presence of specific autoantibodies and endocrine abnormalities through laboratory tests [1-3].

T1DM is characterized by an autoimmune destruction of the ß-cells of the pancreas resulting in the loss of the ability to produce insulin [4]. Patients with T1DM often exhibit a higher prevalence of autoimmune thyroid disease [4]. This association is attributed to a common genetic susceptibility, especially involving human leukocyte antigen (HLA) class II alleles. For example, HLA-DRB103 and HLA-DRB104 are often found in both T1DM and APS3 patients, suggesting a shared genetic predisposition [4,5]. Additionally, the presence of autoantibodies, such as glutamic acid decarboxylase autoantibodies (GAD), is more prevalent in patients with both T1DM and APS3, suggesting a common autoimmune mechanism [4,5]. Patients with APS3 who have T1DM typically present with an older age at diagnosis and more gradual onset, when compared to individuals with only T1DM, indicating different genetic backgrounds [5].

Here we report a case of a type 1 diabetic patient, who developed other autoimmune diseases during his life, meeting the criteria for the diagnosis of APS3.

## Case presentation

Patient was a 64-year-old male, with a history of type 1 diabetes mellitus, diagnosed at the age of 32, presenting microvascular complications, namely diabetic retinopathy and neuropathy. He was referred to a Diabetes consultation in 2014 due to poor metabolic control (hemoglobin A1C 8.8%). An optimized intensive insulin regimen with basal insulin glargine and bolus at all meals with insulin aspart was implemented, improving metabolic control and reaching hemoglobin A1C levels of 7.5%. Also, his T1DM was investigated by measuring antibodies, having tested positive for anti-GAD65, anti-islet cell (ICA) and anti-insulin antibodies. He maintained follow-up for T1DM every six months.

In 2019, the patient exhibited milky-white patches of skin (leukoderma) on the chin, bilateral cervical region, and right hip. He was referred to a dermatology consultation, where he was diagnosed with vitiligo.

In a follow-up appointment in 2020, a blood test showed macrocytic anemia: hemoglobin (Hb) 12.8g/dL, mean corpuscular volume (MCV) 111fL. The rest of the cell lineage was normal (white cell count of 6.4x109/L and a platelet count of 182x109/L). Renal function was normal with a serum creatinine of 0.6 mg/dl. Serum lactate dehydrogenase was normal (199 U/L). Vitamin B12 was decreased (serum concentration of 100 pg/ml) with folic acid at the normal range (14.2 ng/ml). An upper gastrointestinal endoscopy revealed chronic pangastritis. Positive anti-gastric parietal cell antibodies (61.09 U/mL) and negative anti-intrinsic factor antibodies (0.6U/ml) led to a diagnosis of chronic autoimmune gastritis. The patient started monthly injectable vitamin B12 supplementation with normalization of vitamin B12 levels (405 pg/ml) and resolution of anemia (Hb 16.3 g/dL).

Since the patient already had three autoimmune diseases (T1DM, vitiligo, and chronic autoimmune gastritis), it was decided to screen for antibodies of two other autoimmune diseases that are frequently associated with T1DM: autoimmune thyroiditis and celiac disease.

Thyroid antibodies were tested, revealing positive anti-thyroid peroxidase (TPO) antibodies. The patient had slightly elevated thyrotropin (thyroid-stimulating hormone (TSH)) with normal free thyroxine (FT4), indicating subclinical hypothyroidism. The patient did not require supplementation, and remained under surveillance, with annual measuring of TSH and FT4.

Regarding celiac disease, anti-gliadin and anti-transglutaminase antibodies were tested and both were negative. Additionally, adrenocorticotropic hormone (ACTH) and cortisol levels were normal, ruling out adrenal involvement. The results of the laboratory analysis are summarized in Table 1.

**Table 1:** Summary of clinical investigation.

Test	Result	Reference range and units
Anti-glutamic acid decarboxylase (GAD) 65	193	<5.0 U/ml
Anti-insulin (IAA)	1.5	0-0.4 U/ml
Anti-islet cell (ICA)	12.91	<0.7 U/ml
Hemoglobin A1C	7.5%	4.6-6.0%
Hemoglobin	12.8	13.2-16.6 g/dl (man)
Creatinine	0.6	0.5-1.2 mg/dL
Vitamin B12	100	191-663 pg/ml
Folic Acid	14.2	2.2-17.5 ng/ml
Anti-intrinsic factor	0.6	<10 U/ml
Anti-parietal cells	61.09	<25 U/ml
Thyrotropin (TSH)	6.34	0.27-4.20 mIU/L
Free thyroxine (FT4)	16.50	8.24-21.0 pmol/L
Anti-thyroid peroxidase (TPO)	462	<25 UI/ml
Anti-thyroglobulin	33	<40 UI/ml
Adrenocorticotropic Hormone (ACTH)	16.5	<63.3 ng/L
Cortisol	421	171-536 nmol/L
Anti-transglutaminase IgA	0.2	< 7 U/ml
Anti-transglutaminase IgG	0.5	<10 U/mL
Anti-gliadin IgG	0.4	< 7 U/ml

This case demonstrates a type 1 diabetic patient additionally diagnosed with vitiligo, chronic autoimmune gastritis, and chronic autoimmune thyroiditis. Excluding adrenal gland involvement, this constitutes a diagnosis of APS type 3. 

## Discussion

Autoimmune polyglandular syndrome was first reported by Schmidt in 1926 [6], although APS3 was only first described by Betterle et al. in 1980 [7]. This syndrome is characterized by the coexistence of autoimmune thyroid disease with other autoimmune conditions, excluding Addison's disease [1]. The prevalence is estimated to be one in 20,000 individuals [1,7].

This case illustrates the complexity and multifaceted nature of APS3, presenting with four autoimmune diseases, diagnosed at different times in life: chronic autoimmune thyroiditis, T1DM, vitiligo and chronic autoimmune gastritis.

Patients with APS3 and T1DM often present with an older age at diagnosis and a slower progression of the disease compared to those with isolated T1DM [4]. This patient, diagnosed with T1DM at the age of 32, reflects this pattern, with subsequent development of vitiligo, chronic autoimmune gastritis and ultimately chronic autoimmune thyroiditis, fulfilling the criteria for APS type 3.

The natural history of autoimmune disease is characterized by progression from latent and subclinical to clinical stages and is associated with the presence of the specific circulating autoantibodies [7]. This case emphasizes the need for a comprehensive autoimmune evaluation, with regular monitoring of endocrine function and autoantibody levels in patients with T1DM, particularly those who do not follow the typical rapid progression or who develop other autoimmune symptoms. This approach ensures that additional autoimmune diseases are identified and managed early, potentially improving patient outcomes and preventing complications.

## Conclusions

This case highlights the necessity for a broad and thorough evaluation of autoimmune diseases in patients with T1DM and other autoimmune conditions. The interplay between genetic predisposition and the development of multiple autoimmune diseases necessitates a vigilant and proactive approach. By recognizing the potential for multiple autoimmune disorders and implementing regular surveillance and screening for other autoimmune diseases before symptoms appear, healthcare providers can ensure timely diagnosis and optimal management of APS3. This approach not only improves clinical outcomes but also enhances the overall well-being of patients affected by this complex syndrome.
